# Improved sampling scheme for LiDAR in Lissajous scanning mode

**DOI:** 10.1038/s41378-022-00397-9

**Published:** 2022-06-15

**Authors:** Junya Wang, Gaofei Zhang, Zheng You

**Affiliations:** 1 School of Mechanical Science and Engineering, Huazhong University of Science and Technology, 430074 Wuhan, China; 2 Department of Precision Instrument, Tsinghua University, 10084 Beijing, China; 3 State Key Laboratory of Precision Measurement Technology and Instruments, Tsinghua University, 10084 Beijing, China

**Keywords:** Electrical and electronic engineering, Optical sensors

## Abstract

MEMS light detection and ranging (LiDAR) is becoming an indispensable sensor in vehicle environment sensing systems due to its low cost and high performance. The beam scanning trajectory, sampling scheme and gridding are the key technologies of MEMS LiDAR imaging. In Lissajous scanning mode, this paper improves the sampling scheme, through which a denser Cartesian grid of point cloud data at the same scanning frequency can be obtained. By summarizing the rules of the Cartesian grid, a general sampling scheme independent of the beam scanning trajectory patterns is proposed. Simulation and experiment results show that compared with the existing sampling scheme, the resolution and the number of points per frame are both increased by 2 times with the same hardware configuration and scanning frequencies for a MEMS scanning mirror (MEMS-SM). This is beneficial for improving the point cloud imaging performance of MEMS LiDAR.

## Introduction

3D imaging LiDAR consists of a laser, scanning mechanism and detector. At present, the scanning mechanism driven by motors has severely restricted the realization of miniaturization and high performance of LiDAR systems, which makes it difficult to meet the needs of aerospace^1,2^, vehicle^3,4^, and surveying and mapping^5^ applications. With the development of MEMS technology, especially the commercialization of MEMS-SM, the National Institute of Standards and Technology (NIST) proposed the next generation LiDAR technology architecture based on MEMS-SM in 2004^6^. The US Army Research Laboratory^7–14^ configured LiDAR operating parameters to form images of 256 (h) × 128 (v) pixels over a 15 × 7.5° field of view (FOV)^15^. Yeungnam University^16^ developed LiDAR to measure a range image with a resolution of 848 (h) × 480 (v) point locations at the FOV of 43.2 × 24.3°, and the angular resolution is 0.0509° in simulation^17^. Technical University of Denmark^18^, KTH Royal Institute of Technology^19^ have long been committed to the application of MEMS-SM in 3D imaging LiDAR. However, due to the special scanning mode of MEMS LiDAR and the limitation of angular resolution and the number of points per frame, the output points of current LiDAR cannot meet the needs of lane detection^20^, object detection^21,22^, or superresolution^23^.

The nonresonant-driven MEMS-SM supports three scanning modes^24^: point-to-point scanning mode on both axes with the laser beam stopping at each angle, then stepping to the next angle, resonant scanning mode on the *X*-axis and quasistatic on the *Y*-axis, with scanning mode on both axes. All three scanning forms are nonlinear. The spectrum of a quasistatic-driving mode, such as a triangle wave, not only consists of its fundamental frequency but also contains all of its odd harmonics, which is not suitable for high-speed scanning^25,26^. Moreover, spiral and rosette trajectory patterns support scanning mode with both axes^27^. However, the Lissajous trajectory has the special geometric characteristics necessary for the rapid reconstruction of nonrectilinear Cartesian *k*-space trajectories with constant sampling time intervals^28^. The Lissajous scheme is a time-efficient sampling scheme and is currently deployed in various fields. Its application ranges from atomic force microscopy (AFM)^29^, microscopy^30^, electroencephalography reconstruction^31^, frequency-modulated gyroscopes^32^, fast eddy current testing^33^, augmented reality (AR)^34^, and magnetic resonance imaging (MRI)^35^ to MEMS imaging LiDAR^36^.

The horizontal resolution of point cloud sampling of motor-driven mechanical LiDAR is guaranteed by the rotation of the motor, and the vertical resolution is guaranteed by the installation position of the laser, so the uniform space sampling point cloud data can be obtained by equal time interval ranging. The LiDAR point cloud sampling method based on scanning mechanisms, such as galvanometers^37^, pyramid polygon mirrors^38^, and double optical wedges^39^ has not been specially designed. While the new LiDAR based on MEMS-SM, or the imaging equipment using MEMS-SM as the movable part, uses Lissajous scanning during imaging mode, the scan-driving function is a sine curve with two orthogonal axes, so it is difficult to realize grid sampling in time and space. Previous studies^40^ have found two irrelevant parameters that determine the trajectory and proposed a design method of Lissajous trajectories. Jones^41^ and Hoste^42^ analyzed the theoretical feasibility of Lissajous trajectory gridding with knots from the mathematical point of view. Likes^43^ proposed that the Lissajous trajectory was original, and its sampling density was studied by Hardy et al.^44^. Moriguchi^28^ demonstrated that the Lissajous curve has the feasibility of Cartesian gridding of sampling points with constant sampling time intervals, and when $f_x = 11\,{\rm {Hz}}$, $f_y = 9\,{\rm {Hz}}$, and the sampling frequency of the point cloud is 198 Hz, the point cloud satisfies the Cartesian grid. In ref. ^45^, nonrectilinear Cartesian grid sampling was used to capture the statistical dependence of the two remote-sensing images for registration.

To date, the inherent link among the trajectory, the sampling scheme, and the associated complexity of the remeshing process has been investigated to only a limited extent. To improve the utilization of MEMS-SM, the angular resolution and the number of points per frame of LiDAR are improved at the same hardware configuration^4,46^. In this paper, the sampling scheme of MEMS LiDAR in Lissajous scanning mode is studied. First, the corresponding sampling scheme is redesigned in a more compact trajectory^40^. Second, through the theoretical analysis of the sampling scheme satisfying the Cartesian grid, a general sampling scheme of the Lissajous trajectory pattern is proposed.

## Methods

**Fig. 1:**
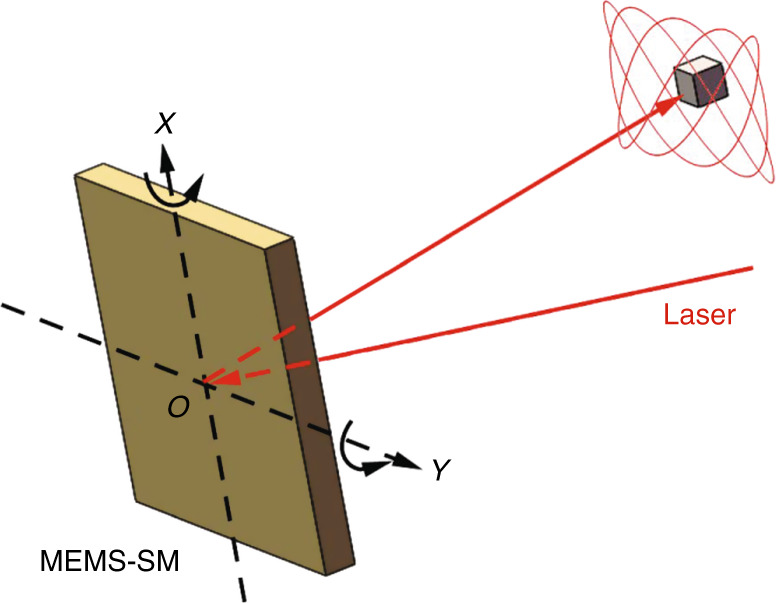
Lissajous trajectory driven by a MEMS-SM

The Lissajous scanning trajectory is driven by two single-tone harmonic waveforms of constant frequency and amplitude, which are usually generated by a 2D MEMS-SM^47^. A schematic diagram is shown in Fig. 1.

**Fig. 2:**
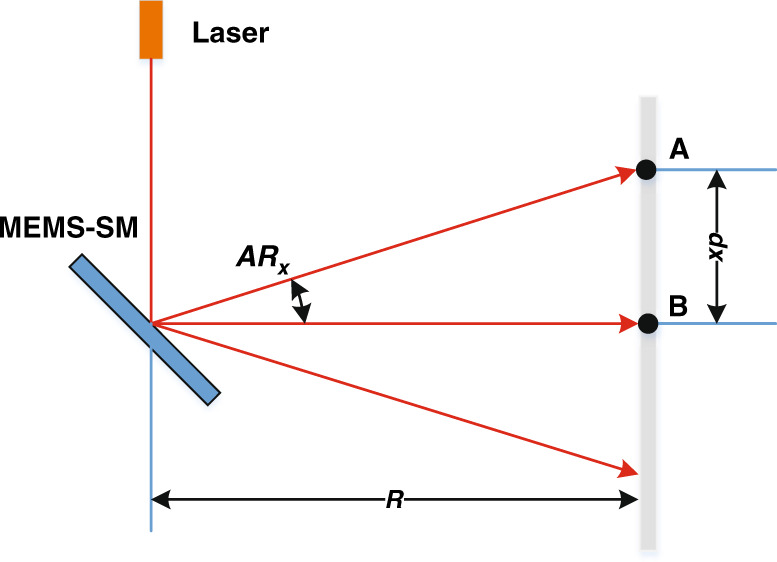
Assuming that the divergence angle of the laser beam is very small, the spot at the target is far smaller than the radar cross section (RCS) of the target. The *X*-axis of angular resolution (AR_*x*_), ${\rm {AR}}_x = \arctan \frac{{{\rm {d}}x}}{R}$, where ${\rm {d}}x$ is the *X*-axis coordinate difference of two adjacent points (point A and point B) in space, and *R* is the distance between the MEMS-SM and the target. In the nonrectilinear trajectory, two adjacent points in space are usually not adjacent in time

According to the imaging principle of LiDAR, the angular resolution of the image can be calculated, and a schematic diagram is shown in Fig. 2.

The sample points are all on the scanning trajectory, so first, the characteristics of the Lissajous trajectory are analyzed, which is composed of two orthogonal cosine curves:

1 $$\left\{ {\begin{array}{*{20}{c}} {X = A_x\cos (2\pi f_xt + \varphi _x)} \\ {Y = A_y\cos (2\pi f_yt + \varphi _y)} \end{array}} \right.$$

where *X* and *Y* are the horizontal and vertical coordinates of scanning points and where $A_x$ and $A_y$ denote the scanning amplitude of the *X*-axis and *Y*-axis directions, respectively. Here, *t* is time, and $f_x,f_y,\varphi _x,\varphi _y$ are the biaxial scanning frequencies and phases of the *X*-axis and *Y*-axis directions, respectively. If $f_x,f_y$ are both integers and a greatest common divisor (GCD) $f_0$ exists, then the following Eq. (2) holds:

2 $$\frac{{f_x}}{{f_y}}{{{\mathrm{ = }}}}\frac{{n_xf_0}}{{n_yf_0}} = \frac{{n_x}}{{n_y}}$$

We assume that point ($x_0,y_0$) is the knot of the Lissajous trajectory, which appears at $t_0$.

3 $$t_0 = \frac{{ \pm \!\arccos \left( {\frac{{x_0}}{{A_{{{\mathrm{x}}}}}}} \right) - \varphi _x}}{{2\pi f_x}} + \frac{l}{{f_x}},\left\{ {\begin{array}{*{20}{c}} {l = 0,1,2, \ldots ,n_x - 1,({\rm {when}}{\kern 1pt} {\kern 1pt} {\kern 1pt} {\kern 1pt} \pm \arccos \left( {\frac{{x_0}}{{{{{\mathrm{A}}}}_{{{\mathrm{x}}}}}}} \right) - \varphi _x \,>\, 0)} \\ {l = 1,2, \ldots ,n_x,({\rm {when}}{\kern 1pt} {\kern 1pt} {\kern 1pt} {\kern 1pt} {\kern 1pt} \pm \arccos \left( {\frac{{x_0}}{{{{{\mathrm{A}}}}_{{{\mathrm{x}}}}}}} \right) - \varphi _x \,<\, 0){\kern 1pt} {\kern 1pt} {\kern 1pt} {\kern 1pt} {\kern 1pt} {\kern 1pt} {\kern 1pt} {\kern 1pt} {\kern 1pt} {\kern 1pt} {\kern 1pt} } \end{array}} \right.$$

Bringing Eq. (3) into Eq. (1), we can obtain $y_l$ in Eq. (4):

4 $$y_l = A_{{{\mathrm{y}}}}\cos \left[ { \pm \frac{{n_y\arccos \left( {\frac{{x_0}}{{A_{{{\mathrm{x}}}}}}} \right)}}{{n_x}} + 2\pi \frac{{n_yl}}{{n_x}} + \left( {\varphi _{{{\mathrm{y}}}} - \frac{{n_y}}{{n_x}}\varphi _x} \right)} \right]$$

We define the phase parameter *k*

5 $$k = \frac{4}{\pi }\left( {n_x\varphi _y - n_y\varphi _x} \right)$$

Therefore, $y_l$ can be simplified to Eq. (6)

6 $$y_l = A_{{{\mathrm{y}}}}\cos \left[ { \pm \frac{{n_y\arccos \left( {\frac{{x_0}}{{A_{{{\mathrm{x}}}}}}} \right)}}{{n_x}} + 2\pi \frac{{n_yl}}{{n_x}} + \frac{{k\pi }}{{4n_x}}} \right]$$

Of all the possible values of *l*, two unequal $l_1$ and $l_2$ ensure $y_{l_1} = y_{l_2}$. Thus, we can obtain Eq. (7)

7 $$\pm 4n_y\frac{{\arccos \left( {\frac{{x_0}}{{A_{{{\mathrm{x}}}}}}} \right)}}{\pi } = 4i - k$$

where *i* is an integer and $\arccos \left( {\frac{{x_0}}{{A_{{{\mathrm{x}}}}}}} \right) \in [0,\pi ]$ is satisfied, the range of *i* is $[0,n_y - 1]$, and bringing Eq. (7) to Eq. (3), the time when the knots appear is shown in Eq. (8).

8 $$t_0 = \frac{{4\pi i - k\pi - 4\pi n_y + 8\pi ln_x}}{{8\pi f_0n_xn_y}}$$

where *i* is an integer. Therefore, we can obtain the time interval Δ between two adjacent points in time.

9 $$\Delta = t_0(i) - t_0(i - 1) = \frac{1}{{2\pi f_0n_xn_y}}$$

## Results and discussion

**Fig. 3:**
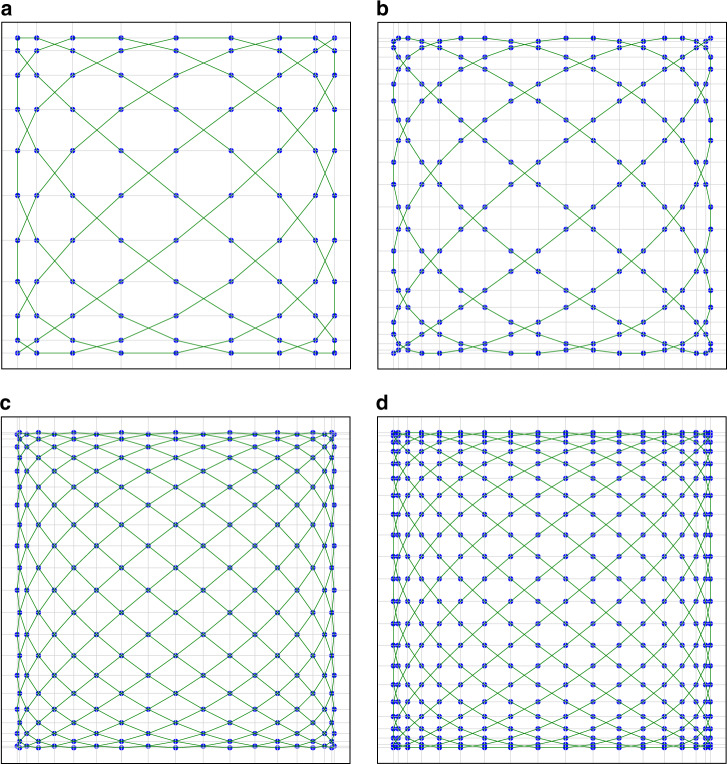
Different trajectories and sampling schemes at the same scanning frequencies of the MEMS-SM $(f_x = 11\,{\bf{Hz}},{{f}}_{{y}} = 9\,{\bf{Hz}})$. The green line is the Lissajous scanning trajectory, and the blue dot is the spatial position of the point. The trajectories and sampling schemes are shown below. **a** $k = 0,f_s = 1/\Delta ,t_1 = 0$, **b** $k = 0,f_s = 2/\Delta ,t_1 = 0$, **c** $k = 2,f_s = 2/\Delta ,t_1 = 0$, and **d** $k = 2,f_s = 2/\Delta ,t_1 = \Delta /4$

A higher sampling rate implies more points and higher resolution. However, in the case of the Cartesian grid, once the trajectory is determined, the matching sampling rate is also determined, and the sampling rate cannot be changed at will. In a Lissajous trajectory period ($1/f_0{\kern 1pt}$), assuming that $f_{\rm {s}}$ is the sampling frequency, the sampling start time is $t_1$ and the end time is $t_2$, the sampling scheme in ref. ^28^ can be summarized as Eq. (10), and the diagram of the sampling point cloud is shown in Fig. 3a.

10 $$\left\{ {\begin{array}{l} {f_s = 1/\Delta = 2n_xn_yf_0}\\ t_{1} = 0\\ t_2 = 1/f_0\end{array}} \right.$$

With the trajectory of ref. ^28^, we increase the sampling frequency by only twice to obtain the point cloud shown in Fig. 3b. The point cloud does not satisfy the Cartesian grid. According to the trajectory design method proposed in ref. ^40^, when the scanning frequency ratio remains unchanged and the phase parameter *k* = 2 is set, the angular resolution of the Lissajous trajectory decreases. At this time, according to the sampling scheme of ref. ^28^, the point cloud shown in Fig. 3c is obtained. We find that the point cloud coincides with the knots of the trajectory and does not meet the Cartesian grid. Therefore, a new sampling scheme is proposed in this paper, that is, when the scanning frequency ratio is unchanged and the phase parameter *k* = 2. The parameters of the proposed sampling scheme are shown in Eq. (11), and the diagram of the sampling point cloud is shown in Fig. 3d.

11 $$\left\{ {\begin{array}{l} {f_s = 2/\Delta = 4n_{x} n_{y} f_0} \\ {t_1 = \frac{1}{{2f_s}} = \frac{1}{{8n_{x} n_{y}f}}} \\ {t_2 = 1/f_0} \end{array}} \right.$$

Through the comparison between Fig. 3, we know that a perfect sampling scheme in the same scanning frequencies has three characteristics: one is the phase parameter *k* = 2^40^. Two is that the sampling interval is equal to the space interval of the trajectory knots. The last is that the starting sampling time is in the middle of the 1st and 2nd knot.

**Fig. 4:**
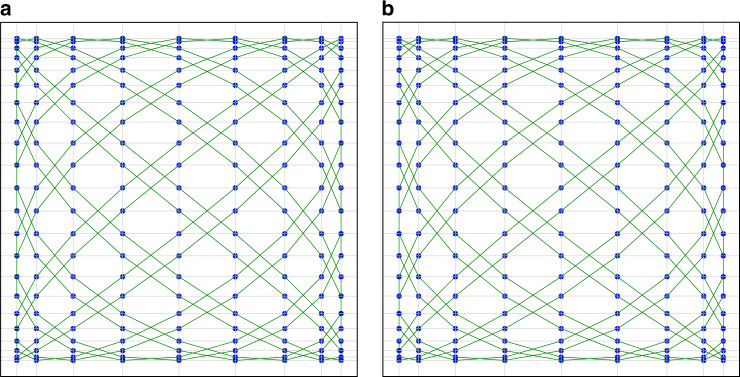
Different trajectories with the improved sampling scheme. **a**, **b** When the scanning frequencies of the two axes of the scanning mirror are $f_{x} = 11\,{\bf{Hz}},{{f}}_{{y}} = 9\,{\bf{Hz}}$, respectively, the trajectories and sampling points under different *k* parametric conditions. The green line is Lissajous scanning trajectory, and the blue dot is the spatial position of the sampling point. The different parametric conditions are **a** $k = 1,f_s = 1/\Delta ,t_1 = 0$ and **b** $k = 3,f_s = 1/\Delta ,t_1 = 0$

The period of *k* is 8^48^, and the trajectories symmetry of the first half period and the second half period. Therefore, this paper takes $k = 0,1,2,3$ as an example. The trajectories are shown in Figs. 3a, 4a, 3d, and 4b. We find that the point cloud is exactly the same when *k* = 1 and *k* = 3, even if they have different trajectories.

By taking the four kinds of trajectories (Figs. 3a, 4a, 3d, 4b) as an example, the improved sampling scheme proposed in this paper is used to make a difference on the grid of sampling points, and the variance of these data is used as the basis for selecting trajectories. Ref. ^26^ is named the scanning density. As an additional finding, the adjacent knots of the trajectories in Fig. 3a and d are identical in time and space. However, the adjacent knots of trajectories in Fig. 4a and b are different in time and space.

**Fig. 5:**
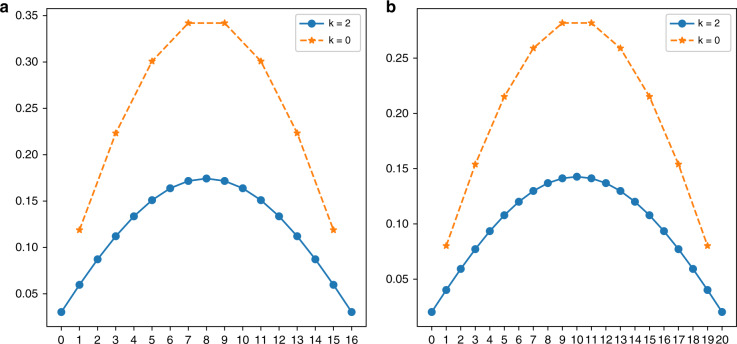
Two-axis grid difference of different trajectories with the improved sampling scheme. When the scanning frequencies of the two axes of the scanning mirror are $f_x = 11\,{\bf{Hz}},{{f}}_{{y}} = 9\,{\bf{Hz}}$, two-axis grid difference of the different trajectories (*k* = 0 or *k* = 2) with the improved sampling scheme are shown. The *X*-axis represents the number of points, and the *Y*-axis represents the grid difference. **a** *X*-axis grid difference of two adjacent points in space. **b** *Y*-axis grid difference of two adjacent points in space

It can be seen from Fig. 3a that when *k* = 0, the point cloud can be divided into 11 × 9 grids, so the difference of the *X*-axis is 8 points and that of the *Y*-axis is 10 points. Similarly, when *k* = 2, there are 17 points on the *X*-axis and 21 points on the *Y*-axis in Fig. 5. When *k* = 1 or *k* = 3, the difference in the *X*-axis is exactly the same as with *k* = 0; moreover, the *Y*-axis is exactly the same as with *k* = 2. The smaller the difference is, the denser the point cloud and the smaller the angular resolution.

Therefore, at the same scanning frequencies of the MEMS-SM ($f_x = 11\ {\rm{Hz}},\,f_y = 9\ {\rm{Hz}}$), the number of points per frame and the angular resolution of the two axes are as shown in Table 1.

**Table 1:** Comparison of three sampling schemes

	Knots	Ref. ^28^	This paper
PPS (pcs)	202	198	396
AR_*x*_ (°)	0.1743	0.3420	0.1743
AR_*y*_ (°)	0.1427	0.2817	0.1427

In Table 1, ${\rm {AR}}_x$ is the angular resolution in the *X*-axis; ${\rm {AR}}_y$ is the angular resolution in the *Y*-axis, and its calculation method is shown in ref. ^49^; PPS represents the effective points per second. We can see clearly that the algorithm performance in this paper increases 2 times when the number of sampling points is 396, and the resolution of both axes is increased by 2 times.

**Fig. 6:**
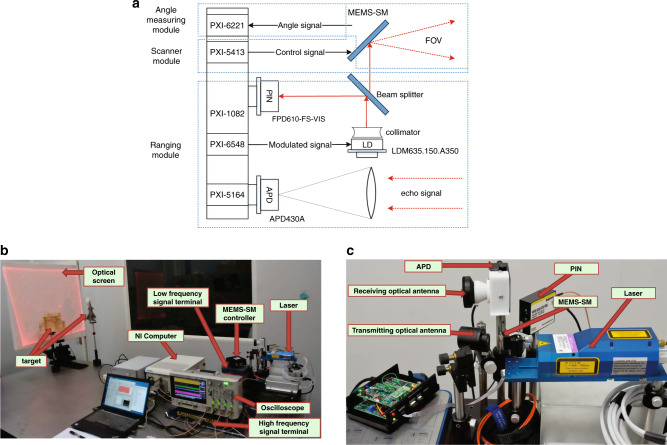
Schematic diagram of the MEMS LiDAR architecture and the experimental platform. **a** Schematic diagram of MEMS LiDAR architecture. **b** Photo of the experimental platform of the MEMS LiDAR. **c** Photo of the laser and the receiver. The experimental equipment uses NI chassis (PXI-1082) to load computer module. The pulse transmitting module (PXI-6584) generates pulses to modulate the laser (LDM635.150.A350) amplitude. Through a beam splitter, part of the light is received by PIN (FPD610-FS-VIS), and part of the light is reflected by MEMS-SM and irradiates the target surface. The diffuse reflection light is received by APD (APD430A). The analog signals generated by PIN and APD are synchronously collected by PXI-5164 and used to calculate the time of fight and then measure the distance. The signal of Lissajous scanning trajectory is generated by signal generator PXI-5413 and controller of MEMS-SM driving module. The angle measurement is completed by PXI-6221. So far, the angle and distance measurement synchronization signal are generated by Field Programmable Gate Array (FPGA) in PXI-5164

To verify the trajectory and sampling scheme in Fig. 3d, a MEMS LiDAR prototype is designed for imaging experiments, and its structure principle is shown in Fig. 6.

**Fig. 7:**
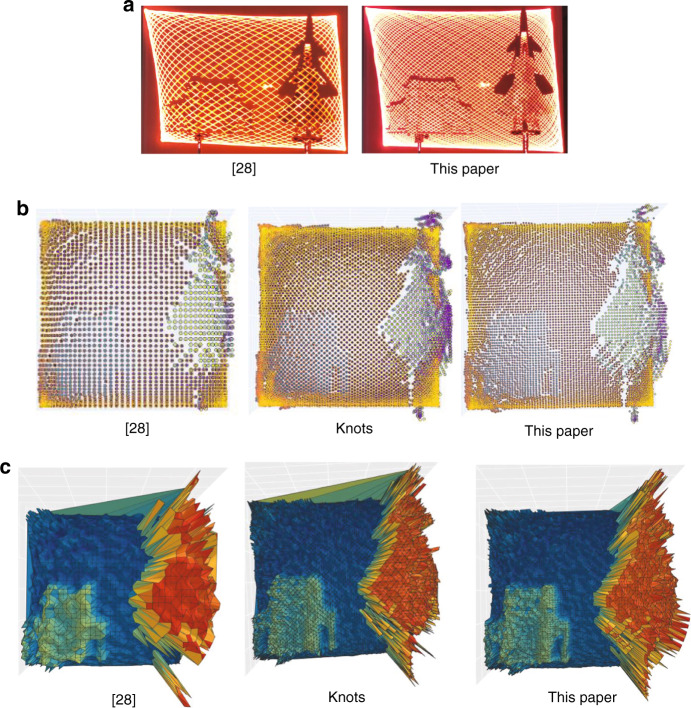
The range images based on different sampling schemes. The scanning trajectories in ref. ^28^ and designed in this paper are used to scan and image the target models respectively. The photos of the different trajectories and targets are shown. The targets are the models of the Tsinghua University Second Gate and the J-20 fighter. Three sampling schemes are used for imaging. One sampling scheme in ref. ^28^ is based on the scan trajectory designed in ref. ^28^. The other two sampling schemes are based on the scanning trajectory designed in this paper. **a** Targets and two scanning trajectories. **b** Point cloud obtained by different sampling schemes. **c** 3D front view images obtained by different sampling schemes

In the experiment, the targets are the Second Gate model of Tsinghua University, which is made of light-colored wood with a reflectivity of approximately 0.8 and a size of 300 × 65 × 293 mm, and the J-20 fighter model, which is made of dark camouflage engineering plastic with baking paint printing. Part of the surface is similar to specular reflection, with a reflectivity of approximately 0.2 and a size of 210 × 14 × 13 mm, including transparent plastic of the cockpit, with a size of approximately 10 × 5 mm. For example, the parameters of the Lissajous scanning trajectory are:$n_x:n_y = 53:51$, the FOV of the MEMS-SM is 10 × 10°, and after amplification by the transmitting antenna, the optical field of view FOV = 30 × 30°, $f_0 = 10{\kern 1pt} {\kern 1pt} \rm{Hz}$. The results are shown in Fig. 7.

From the point cloud data, the outlines of the Second Gate model and J-20 model can be clearly distinguished. The scanning frequencies are $f_x = 530{\kern 1pt} {\kern 1pt} {\rm {Hz}}$ and $f_y = 510{\kern 1pt} {\kern 1pt} {\rm {Hz}}$. The number of points per frame obtained by the sampling scheme in ref. ^28^ is 5406, and the angular resolution is ${\rm {AR}}_x = 0.5563^\circ$ and ${\rm {AR}}_y = 0.6119^\circ$. The number of point clouds per frame obtained by the sampling scheme by knots is 5510, and the angular resolution is ${\rm {AR}}_x = 0.2781^\circ$ and ${\rm {AR}}_y = 0.3060^\circ$. The number of points per frame obtained by the sampling scheme proposed in this paper is 10,812, and the angular resolution is ${\rm {AR}}_x = 0.2781^\circ$ and ${\rm {AR}}_y = 0.3060^\circ$.

At the same hardware configuration, achieving higher angular resolution and more points per frame is the main work of this paper. Therefore, this paper first designs a better sampling scheme in the specific Lissajous trajectory and then summarizes the general sampling scheme to solve the coupling problem of Lissajous trajectory, sampling scheme and gridding. In the same MEMS-SM and the same scanning frequencies, the angular resolution and the number of points per frame are increased by 2 times (simulation in Table 1, experiment in Fig. 7b), and the effect of 3D reconstruction is better (Fig. 7c). A MEMS LiDAR prototype demonstration system is built to test the effectiveness of the improved method. However, we find that the sampling frequency we set in Fig. 3a is$2f_0n_xn_y = 198{\kern 1pt} {\kern 1pt} {\rm {Hz}}$, but only 99 sampling points are found in Fig. 3a. This is because the other 99 sampling points completely coincide with the 99 sampling points; that is, the image frame frequency is 2 Hz. Even so, the conclusion of Table 1 is still true.

## Conclusion

For the imaging of a Lissajous trajectory system, such as with MEMS LiDAR, the sampling scheme of the point cloud not only is related to the trajectory but also needs to match the Cartesian grid. Therefore, it is difficult to utilize a universal sampling scheme. This paper establishes a functional relationship between the time and space parameters of point clouds so that the points sampled in equal time coincide with the Cartesian grid. Compared with the existing methods, the sampling scheme in this paper is more general, and the theoretical resolution performance of point cloud images is better.

## Supplementary information

Graphical abstract
